# Neutrophil Extracellular Traps Contribute to COVID-19 Hyperinflammation and Humoral Autoimmunity

**DOI:** 10.3390/cells10102545

**Published:** 2021-09-26

**Authors:** Jiram Torres-Ruiz, Abdiel Absalón-Aguilar, Miroslava Nuñez-Aguirre, Alfredo Pérez-Fragoso, Daniel Alberto Carrillo-Vázquez, José Luis Maravillas-Montero, Nancy R. Mejía-Domínguez, Luis Llorente, Beatriz Alcalá-Carmona, Jaquelin Lira-Luna, Carlos Núñez-Álvarez, Guillermo Juárez-Vega, David Meza-Sánchez, Thierry Hernández-Gilsoul, Miguel Tapia-Rodríguez, Diana Gómez-Martín

**Affiliations:** 1 Department of Immunology and Rheumatology, Instituto Nacional de Ciencias Médicas y Nutrición Salvador Zubirán, Mexico City 14080, Mexico; josetorresruiz85@gmail.com (J.T.-R.); abdiel_chapiz28@hotmail.com (A.A.-A.); minulaaa123@gmail.com (M.N.-A.); peralfra07@hotmail.com (A.P.-F.); luisllorentepeters57@gmail.com (L.L.); amarielith@gmail.com (B.A.-C.); jaquelin.lira.luna@gmail.com (J.L.-L.); nuac80df@gmail.com (C.N.-Á.); 2 Emergency Medicine Department, Instituto Nacional de Ciencias Médicas y Nutrición Salvador Zubirán, Mexico City 14080, Mexico; thierry.hernandezg@incmnsz.mx; 3 Department of Internal Medicine, Instituto Nacional de Ciencias Médicas y Nutrición Salvador Zubirán, Mexico City 14080, Mexico; danielbeatle94@gmail.com; 4 Red de Apoyo a la Investigacion, Coordinacion de Investigación Científica, Universidad Nacional Autónoma de México, Mexico City 04510, Mexico; maravillas@cic.unam.mx (J.L.M.-M.); nnmejia@cic.unam.mx (N.R.M.-D.); gumo.vega@gmail.com (G.J.-V.); dmeza@cic.unam.mx (D.M.-S.); 5 Microscopy Unit, Instituto de Investigaciones Biomédicas, Universidad Nacional Autónoma de México, Mexico City 04510, Mexico; mtapia@biomedicas.unam.mx

**Keywords:** COVID-19, SARS-CoV-2, NETs, LDG, LL-37, HMGB1, ISG-15, DNA-complex, DNase and autoimmunity

## Abstract

The coronavirus disease 2019 (COVID-19) is related to enhanced production of NETs, and autoimmune/autoinflammatory phenomena. We evaluated the proportion of low-density granulocytes (LDG) by flow cytometry, and their capacity to produce NETs was compared with that of conventional neutrophils. NETs and their protein cargo were quantified by confocal microscopy and ELISA. Antinuclear antibodies (ANA), anti-neutrophil cytoplasmic antibodies (ANCA) and the degradation capacity of NETs were addressed in serum. MILLIPLEX assay was used to assess the cytokine levels in macrophages’ supernatant and serum. We found a higher proportion of LDG in severe and critical COVID-19 which correlated with severity and inflammatory markers. Severe/critical COVID-19 patients had higher plasmatic NE, LL-37 and HMGB1-DNA complexes, whilst ISG-15-DNA complexes were lower in severe patients. Sera from severe/critical COVID-19 patients had lower degradation capacity of NETs, which was reverted after adding hrDNase. Anti-NET antibodies were found in COVID-19, which correlated with ANA and ANCA positivity. NET stimuli enhanced the secretion of cytokines in macrophages. This study unveils the role of COVID-19 NETs as inducers of pro-inflammatory and autoimmune responses. The deficient degradation capacity of NETs may contribute to the accumulation of these structures and anti-NET antibodies are related to the presence of autoantibodies.

## 1. Introduction

The severe acute respiratory syndrome coronavirus 2 (SARS-CoV-2) infection has been related to exuberant harming autoinflammation and autoimmune responses, especially in critical COVID-19 (Coronavirus Disease 2019) patients [1]. These autoreactive responses share parallel traits with systemic autoimmune and autoinflammatory diseases [2]. Activated neutrophils and macrophages are the hallmark of severe COVID-19 [3], based on differential transcriptomic profiles [4]. During SARS-CoV-2 infection, the type I interferon (IFN) and IL-6 synthesis are dysregulated in myeloid cells [5,6], resembling the type I IFN signature in systemic lupus erythematosus (SLE) [7,8] and the IL-6-mediated macrophage activation syndrome [9]. In COVID-19, neutrophils have been implicated in alveolar damage and capillaritis through the production of neutrophil extracellular traps (NETs), which are webs of DNA decorated by proteins [10]. A neutrophil subset prone to the production of NETs, the low density granulocytes (LDG), display a proinflammatory cytokine release profile [11] and produce NETs resistant to DNase degradation in SLE patients [12]. Enhanced production and deficient clearance of NETs [13] are key drivers of tissue damage in autoimmune and infectious diseases [14], since NETs perpetuate inflammation in SLE [15] and in acute respiratory distress syndrome (ARDS) patients [16]. Sera from critically ill COVID-19 patients induce the release of NETs [17] and contain circulating NET complexes, which correlate with inflammation, severity and immune mediated microthrombi [18]. Moreover, it has been hypothesized that the NET protein cargo in COVID-19 could differentially activate immune cells [19,20] nevertheless, this has not been fully addressed. In severe COVID-19, extrafollicular B cell activation and antibody-secreting cell expansion (previously shown in SLE) correlate with inflammatory markers, multiorgan failure and death [21,22]. Autoantibodies recognizing type I IFN [23], DNA, proteinase-3, myeloperoxidase (MPO), phospholipids and prothrombin [24], have been observed in patients with COVID-19. Some of these antigens are present in NETs and are the targets of anti-nuclear antibodies (ANA) and anti-neutrophil cytoplasmic antibodies (ANCA). Therefore, the enhanced production of NETs may be one of the drivers of autoimmune responses in COVID-19. The aim of the present work is to address the production of NETs by different neutrophil subsets, their protein cargo and their role as inducers of macrophage and autoimmune responses in COVID-19 patients.

## 2. Materials and Methods

This cross-sectional study was conducted at the Instituto Nacional de Ciencias Médicas y Nutrición Salvador Zubirán, a reference care center for patients with COVID-19 in Mexico. We included 82 patients with COVID-19, confirmed with a positive real time polymerase chain reaction (RT-PCR) for SARS-CoV-2 in nasopharyngeal swab and 10 SARS-CoV-2 negative healthy donors. COVID-19 severity was classified according to the following definitions [25]:

1. Mild/moderate disease: Fever, upper respiratory infection symptoms, with or without pneumonia.
2. Severe: Any of the following: respiratory failure, respiratory rate ≥ 30 breaths per minute, oxygen saturation at rest ≤ 93%, PaO_2_/FiO_2_ ≤ 300 mmHg.
3. Critical: Any of the following: need for invasive mechanical ventilation (IMV), shock, multiple organ failure.

Twenty-seven patients (32.92%) had mild/moderate disease, 27 (32.92%) were severe and 28 (34.14%) were critical. Patients with cancer, autoimmune diseases, pregnancy, puerperium, chronic viral diseases and acute bacterial and fungal infections were excluded. A complete medical history, laboratory tests and a low-radiation dose lung computed tomography were performed in patients before the pharmacological treatment was initiated at the emergency department. The protocol was approved by the institutional Ethics and Research committees (REF: 3341) in accordance with the Helsinki declaration. All patients and controls signed an informed consent before inclusion. In order to unveil the role of NETs in COVID-19 hyperinflammation and autoimmunity, the following procedures were performed:

### 2.1. Assessment of the Proportion of LDG by Multiparametric Flow-Cytometry

A total of 60 mL of venous peripheral blood were collected in ethylenediaminetetraacetic acid (EDTA) tubes and separated by density gradients after centrifugation with 60 mL of Ficoll-Paque (GE Healthcare Life Sciences, Chicago, IL, USA) to isolate peripheral blood mononuclear cells (PBMCs). The cells were washed twice with PBS and stained with the zombie aqua viability marker (Biolegend, San Diego, CA, USA). After washing the PBMCs twice with 5% fetal bovine serum (FBS) in PBS, the cells were stained with the following fluorochrome-coupled antibodies: mouse anti human CD14 (cat: 325632), mouse anti human CD15 (cat: 323004) and mouse anti human CD10 (cat: 312204) (all from Biolegend, San Diego, CA, USA). LDG were defined as those cells positive for CD15 and negative for CD14 in the PBMCs pool. CD10 was used as a maturity marker. One million events were acquired in a four laser LSR Fortessa flow cytometer (BD Biosciences, Franklin Lakes, NJ, USA). The absolute numbers of LDG were calculated considering the total leukocytes in a complete blood count drawn the same day. The data were analyzed with the Flow-Jo software v10.7.

### 2.2. Assessment of Plasmatic Circulating NETs and Their Protein Cargo

Plasma circulating NETs, measured as the neutrophil elastase (NE)-DNA complexes were assessed as previously described [26]. Briefly, high binding 96 well plates were coated with 1:2000 mouse anti human neutrophil elastase (Cat: 481001, Calbiochem, San Diego, CA, USA) overnight (ON) at 4 °C. The plates were washed three times with 0.05% Tween/PBS and the non-specific binding sites were blocked with 1% bovine serum albumin (BSA) (MP Biomedicals, Solon, OH, USA) for 6 h. The plasma samples were diluted 1:10 in 1% BSA, 100 mcl of the mix were added to the plate and left incubating ON at 4 °C. The plates were washed three times with 0.05% Tween/PBS and incubated with the anti-DNA antibody conjugated with peroxidase (POD) from the cell death detection kit (Roche, Basilea, Swiss) for 1 h at RT. After washing the plates five times with 0.05% Tween/PBS, the tetramethylbenzidine (TMB) substrate (Thermofisher Scientific, Waltham, MA, USA) was added. The reaction was finalized by the addition of stop solution and the plates were read at 450 nm. Plasma levels of interferon stimulated gene 15 (ISG-15)-DNA, LL-37-DNA, high mobility group box 1 (HMGB1)-DNA complexes were detected as previously described [27]. Briefly, high binding 96 well plates were coated ON at 4 °C with the following 1:100 capture antibodies diluted in coating buffer (Roche, Basilea, Swiss): rabbit anti human ISG-15 (Cat: AP1150a, Abgent, San Diego, CA, USA), mouse anti human LL-37 (Cat: 166770, Santacruz Biotechnology, Dallas, TX, USA), mouse anti human HMGB1 (Cat: 56698, Santacruz Biotechnology, Dallas, TX, USA). The plates were washed three times with 0.05% Tween/PBS, blocked during 1 h at RT with 1% BSA and washed three times with 0.05% Tween/PBS. After incubation with the anti-human DNA antibody conjugated with peroxidase (POD) (Roche, Basilea, Swiss) during one hour at RT, the plates were washed five times with 0.05% Tween/PBS, developed with the TMB substrate (Thermofisher Scientific, Waltham, MA, USA) and read at 450 nm after stop solution was applied.

### 2.3. Quantification of the Amount of LDG and NDG Derived NETs and Their Protein Cargo by Confocal Microscopy

Two million LDG or NDG were resuspended in 1 mL of incubation buffer (RPMI without phenol red, 1% FBS, 1% 10 mM HEPES) and were seeded on poly-L-lysine (Thermofisher Scientific, Waltham, MA, USA) coated coverslips. The neutrophils were incubated for 40 min at 37 °C with 5% CO_2_ and were fixed overnight at 4 °C with 4% paraformaldehyde (Merck, Kenilworth, NJ, USA). Afterwards, the samples were washed three times with PBS, permeabilized with 0.2% triton X-100 for 10 minutes and the non-specific binding sites were blocked with 0.2% gelatin from pork skin (Merck, Kenilworth, NJ, USA) at room temperature (RT) for 30 min. The samples on coverslips were incubated at 37 °C for two hours with the following primary antibodies: rabbit anti human interferon-stimulated gene 15 (ISG-15) (Cat: AP1150a, Abgent, San Diego, CA, USA), rabbit anti human myeloperoxidase (MPO) (Cat: A0398, Agilent, San Diego, CA, USA), mouse anti human LL37 (Cat: 166770, Santacruz Biotechnology, Dallas, TX, USA) and mouse anti human high mobility group box protein 1 (HMGB1) (Cat: 56698, Santacruz Biotechnology, Dallas, TX, USA). After three washes with PBS, the samples were incubated with the following secondary antibodies: donkey anti-rabbit Alexa Fluor 555 (Cat: A32794) and donkey anti-mouse Alexa Fluor 488 (Cat: A32766) (both from Thermofisher Scientific, Waltham, MA, USA). The coverslips were mounted on slides with the ProLong Gold antifade mountant with DAPI (Thermofisher Scientific, Waltham, MA, USA). The images were acquired using an Eclipse Ti-E Nikon confocal microscope (Minato, Tokyo, Japan). The percentage of NETs was quantified dividing the number of structures in which MPO-DNA were co-localized by the number of nuclei in eight 40X fields [28]. The length of the NETs was calculated using the SNT plugin from Fiji as previously described [29]. The expression of ISG-15, LL-37 and HMGB1 was assessed by tracing polygons around the NETs avoiding the cellular bodies and calculating the mean fluorescence intensity with the Fiji software.

### 2.4. Obtention of NETs from LDG and NDG

To isolate LDG, PBMCs were washed twice with MACS buffer and were incubated for 30 min at 4 °C with the anti-human CD66b antibody conjugated to biotin from the CD66b MicroBeads Isolation Kit (Miltenyi Biotec, North Rhine-Westphalia, Germany). Afterwards, the cells were incubated at 4 °C with the anti-biotin microbeads and passed through a LS column to obtain the enriched LDG by positive selection [30]. Viability and purity were above 90% as addressed by trypan blue staining and flow cytometry, respectively. Normal density granulocytes (NDG) were isolated by dextran sedimentation. After LDG and NDG isolation, we observed that the latter were more abundant. Therefore, we resuspended the cells in RPMI without phenol red (Thermofisher Scientific, Waltham, MA, USA), seeded 5–10 million of NDG per well in 12 well plates and 2 million LDG per well in 24 well plates. The cells were incubated for 6 h at 37 °C with 5% CO_2_. The supernatant of the neutrophils was carefully aspirated and NETs were obtained from the bottom of the well after treatment with micrococcal nuclease (Thermofisher Scientific, Waltham, MA, USA) as previously described [28]. The NETs were stored at −80 °C. Since the initial numbers of LDG and NDG were different due to their relative abundance, we quantified the amount of protein in the NETs lysates using the bicinchoninic acid assay (Thermofisher Scientific, Waltham, MA, USA). Fifty micrograms of NETs were used to stimulate the macrophages as described below.

### 2.5. Stimulation of Monocyte-Derived Macrophages with Neutrophil Extracellular Traps and Assessment of Cytokine/Chemokine Production

Peripheral blood monocytes were isolated by positive magnetic separation with LS columns (Miltenyi Biotec, North Rhine-Westphalia, Germany) using CD14 magnetic beads (Miltenyi Biotec, North Rhine-Westphalia, Germany) according to the instructions of the manufacturer. Two million monocytes were re-suspended in RPMI with phenol red (Thermofisher Scientific, Waltham, MA, USA) supplemented with 10% FBS. Afterwards, the monocytes were seeded in 24 well plates and were differentiated into macrophages with M-CSF stimulation (R&D Systems, Minneapolis, MN, USA) as previously described [31]. After 7 days of differentiation, we stimulated the macrophages with 50 micrograms of NETs for six hours and the supernatants were stored at −80 °C until further analysis. The concentration of 29 cytokines were evaluated in the supernatants and sera using the MILLIPLEX Multi-Analyte Profiling (MAP) Human Cytokine/Chemokine Magnetic Bead Panel 29-plex kit (EMD Millipore, Burlington, MA, USA).

### 2.6. Assessment of the Serum Degradation of NETs

Three million neutrophils from healthy donors were stimulated with 2.5 μM phorbol myristate acetate (PMA) (Merck, Kenilworth, NJ, USA) on poly-L-lysine coated coverslips for 4 h to induce NET release. The samples were then incubated ON at 37 °C and 5% CO_2_ with 10% serum from COVID-19 patients or healthy donors diluted in RPMI without phenol red. After fixation for 24 h with 4% PFA at 4 °C, the samples were stained with rabbit anti human MPO as described above. To calculate the percentage of NET degradation, we quantified the amount of NETs remaining after the incubation with 10% serum with or without the addition of 1:10,000 micrococcal nuclease (Thermofisher Scientific, Waltham, MA, USA) or human recombinant DNase (1 U/mL) (Sigma-Aldrich, San Luis, MO, USA) in comparison to the PMA-induced NET formation.

### 2.7. Appraisal of Anti-NET Antibodies, Antinuclear Antibodies (ANA) and Anti-Neutrophil Cytoplasmic Antibodies (ANCA)

Anti-NET IgG antibodies were detected by ELISA in plasma samples, as previously described [32]. Briefly, a flat 96 well high-binding ELISA plate (Corning, Corning, NY, USA) was coated ON at 4 °C with the NET lysate from healthy donor neutrophils stimulated with 2.5 μM PMA at a concentration of 10 µg/mL in 0.05 M bicarbonate buffer for 12 h. Plasma samples were diluted to 1% in blocking buffer (4% BSA) (MP Biomedicals, Solon, OH, USA). After one wash with 0.05% Tween 20/PBS (Thermofisher Scientific, Waltham, MA, USA), samples were added to the plate and incubated in blocking buffer for 120 min at 37 °C. Afterwards, the plate was washed five times with 0.05% Tween 20/PBS (Thermofisher Scientific, Waltham, MA, USA) and incubated with anti-human IgG-HRP (1:10,000) (Cat. ab6759, Abcam, Cambridge, UK) for 90 min at 37 °C. After washing five times with Tween 20/PBS, TMB substrate (Thermofisher Scientific, Waltham, MA, USA) was applied. The reaction was finalized by the addition of stop solution. The plate was read at 450 nm and the optic density index (ODI) for each sample was calculated as previously described [32]. We also detected anti-NETs antibodies by immunofluorescence using 10% sera from COVID-19 patients or healthy donors as previously reported [33]. ANA and ANCA were assessed by indirect immunofluorescence according to international consensus [34].

## 3. Statistical Analysis

Quantitative variables were expressed as medians and interquartile ranges (IQR). Differences between medians were assessed using the Kruskal–Wallis and Mann–Whitney U tests. Correlations between quantitative variables were addressed with the Spearman Rho test with the Bonferroni correction and depicted as a correlation matrix. Association between qualitative variables was addressed using the Chi-square test. The statistical analysis was performed with the support of the GraphPad Prism version 9.0.2 for Mac (San Diego, CA, USA) and the R project software (R Core Team (2021, R: A language and environment for statistical computing. R Foundation for Statistical Computing, Vienna, Austria. URL http://www.R-project.org/).

## 4. Results

Immature LDG are the hallmark of severe and critical COVID-19. 

The percentage and absolute number of total, mature and immature LDG were higher in patients with severe and critical COVID-19 (Table 1 and Supplementary Figure S1).

**Figure 1:**
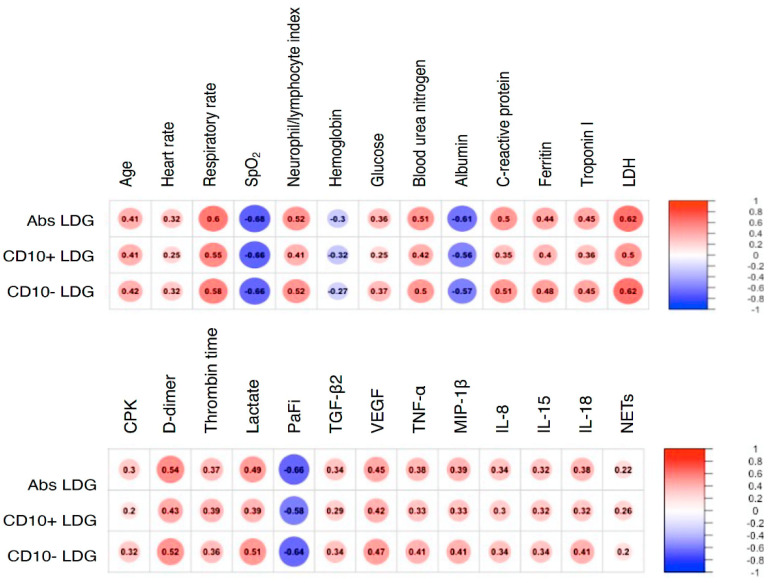
LDG subsets correlate with different biological features of COVID-19. A correlation matrix is depicted with all statistically significant variables. Correlations were addressed using the Spearman Rho with Bonferroni correction. LDG: low density granulocytes. SpO_2_: peripheral oxygen saturation. LDH: lactate-dehydrogenase. CPK: creatine phosphokinase. PaFi: the ratio of the arterial partial pressure of oxygen (“PaO_2_”) from the Arterial Blood Gas divided by the fraction of inspired oxygen (FiO_2_).

LDG from patients with COVID-19 had a predominantly immature phenotype as shown by their absent expression of CD10. Furthermore, as shown in Figure 1 and supplementary Figure S2, LDG subsets correlated with distinctive clinical features of COVID-19.

Interestingly, we found a moderate negative correlation between LDG and PaFi (PaO2/FiO2) and positive correlations with features of tissue turnover such as creatine phosphokinase (CPK), lactate dehydrogenase (LDH), Troponin I, and lactate as well as with variables related to immunothrombosis such as D-dimer and pro-thrombin time. Additionally, LDG had a positive correlation with serum TGF-β2, VEGF, TNF-α, MIP-1β, IL-8, IL-15, IL-18 and plasmatic NETs measured as NE-DNA complexes (Figure 1 and supplementary Figure S2). 

The clinical features of COVID-19 patients and the serum levels of the cytokines and chemokines according to COVID-19 severity are depicted in supplementary Tables S1 and S2, respectively, and displayed as dot plots in supplementary Figures S3 and S4, respectively. 

Circulating NETs containing ISG-15, LL-37, and HMGB1 are detected in patients with COVID-19.

As shown in Table 1 and supplementary Figure S1, patients with critical COVID-19 had a higher amount of circulating NE-DNA and HMGB1-DNA complexes. Additionally, we found a higher amount of circulating LL-37-DNA complexes in severe COVID-19 patients (Table 1 and supplementary Figure S1). Conversely, circulating ISG-15-DNA complexes were lower in patients with severe COVID-19.

NDG from COVID-19 patients are the main source of spontaneous NETs, which carry a distinctive protein cargo.

**Figure 2:**
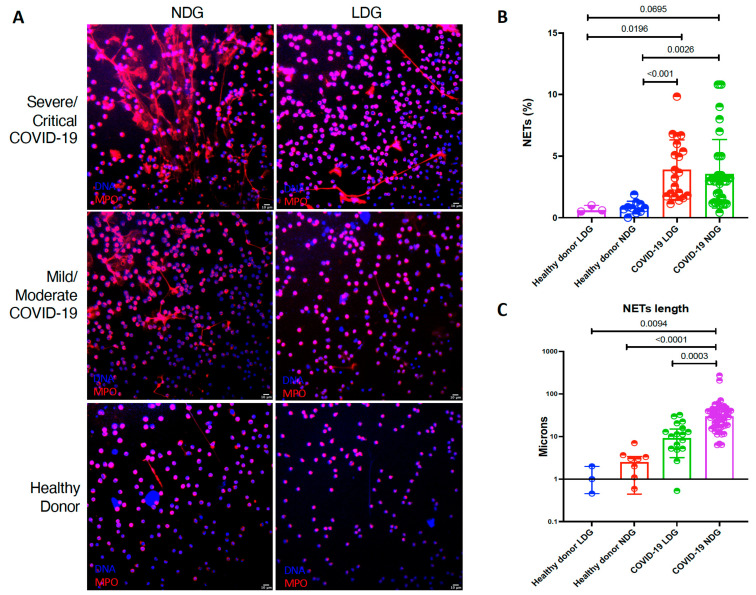
LDG and NDG from COVID-19 patients are prone to spontaneous NET release. (**A**). Representative confocal microscopy image of the spontaneous NETs from COVID-19 neutrophil subsets according to disease severity. (**B**). LDG (*n* = 20) and NDG (*n* = 18) from COVID-19 patients have a higher percentage of NETs in comparison to healthy donor LDG (*n* = 3) and NDG (*n* = 10). (**C**). NETs secreted by NDG from COVID-19 patients have a higher length in comparison to COVID-19 LDG and healthy donor both NDG and LDG. Medians were compared using the Kruskal–Wallis test with Dunn’s multiple comparison test. The length of NETs is expressed in a logarithmic scale.

We evaluated the capacity of both NDG and LDG to produce NETs *ex vivo*. Both LDG and NDG from COVID-19 patients produced a higher percentage of NETs in comparison to healthy donors (Table 2 and Figure 2). Moreover, as shown in Table 2 and Figure 2, NETs from NDG are characterized by a greater length (*p* < 0.0001). 

**Figure 3:**
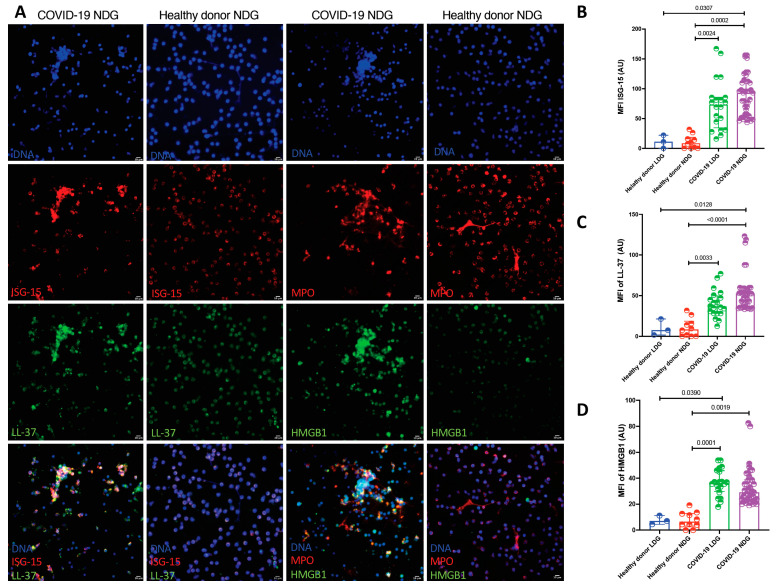
LDG and NDG from COVID-19 patients carry a distinctive pro-inflammatory protein cargo. (**A**). Representative confocal microscopy image showing a higher expression of ISG-15, LL-37 and HMGB1 in NDG NETs from patients with COVID-19 in comparison to healthy donors. (**B**–**D**). Cumulative statistics show a higher MFI of ISG-15, LL-37 and HMGB1 in LDG (*n* = 20) and NDG (*n* = 18) NETs from COVID-19 patients in comparison to NETs from healthy donors LDG (*n* = 3) and NDG (*n* = 10). Differences among medians were addressed with the Kruskal–Wallis test and the multiple comparison Dunn’s test.

When we evaluated the NET protein cargo by confocal microscopy, LDG and NDG from COVID-19 patients showed a higher expression of HMGB1, ISG-15 and LL-37 in comparison with healthy donors (Table 2 and Figure 3).

Sera from severe and critical COVID-19 patients have a deficient degradation of NETs, which is corrected after the supplementation with recombinant human DNase.

**Figure 4:**
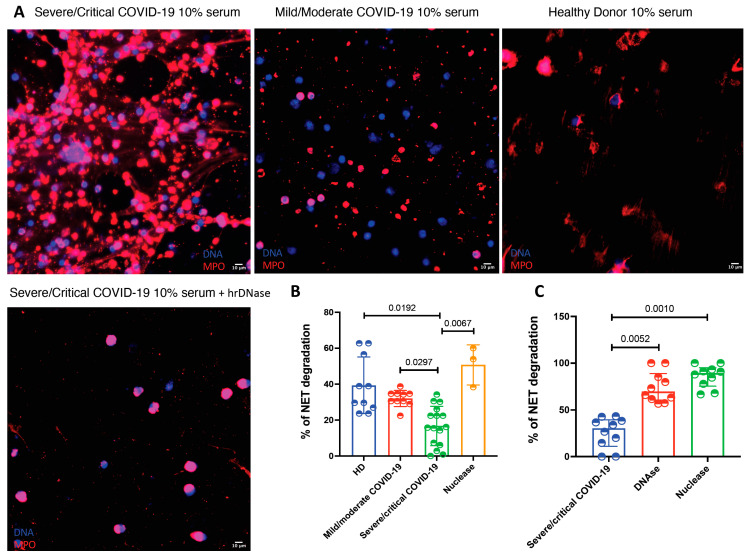
The sera from patients with severe and critical COVID-19 have a deficient NET degrading capacity. (**A**). Representative confocal microscopy images of the NET degradation capacity of COVID-19 sera according to disease severity. Neutrophils from healthy donors (HD) were stimulated with PMA to induced NET formation and then incubated with 10% sera from COVID19 patients or HD. The deficient degradation capacity is corrected after the addition of human recombinant DNAse. B-C. Severe/critical COVID-19 patients (*n* = 16) have a deficient degradation of NETs in comparison to mild/moderate COVID-19 (*n* = 8) and healthy donors (*n* = 10) (**B**). The serum deficient degradation capacity of NETs observed in severe/critical COVID-19 patients (*n* = 16) is reverted after the addition of hrDNase and micrococcal nuclease (**C**). Medians were compared using the Kruskal–Wallis test and Dunn’s multiple comparison test.

A possible mechanism explaining NET accumulation in tissues from COVID-19 patients is a deficient degradation of these structures. Therefore, we decided to test if patients with this viral infection have a deficient degradation capacity of NETs in serum. As depicted in Figure 4, sera from patients with severe/critical COVID-19 had a lower NET degradation capacity in comparison with sera from mild/moderate COVID-19 patients and healthy donors as well as in comparison with micrococcal nuclease treatment (15.98 (6.42–25.20) vs. 34.29 (26.12–58.11) vs. 32.45 (29.39–34.69) and 53.88 (38.88–60.00), respectively, *p* = 0.0004). Since we found that sera from patients with severe and critical COVID-19 had deficient NET degradation capacity, we decided to test if this could be reverted by the addition of hrDNase and micrococcal nuclease. As shown in Figure 4, the addition of DNase was able to restore the degradation capacity of COVID-19 sera (*p* < 0.0001).

Patients with COVID-19 display autoimmunity features including anti-NET antibodies related to ANA and ANCA positivity.

**Figure 5:**
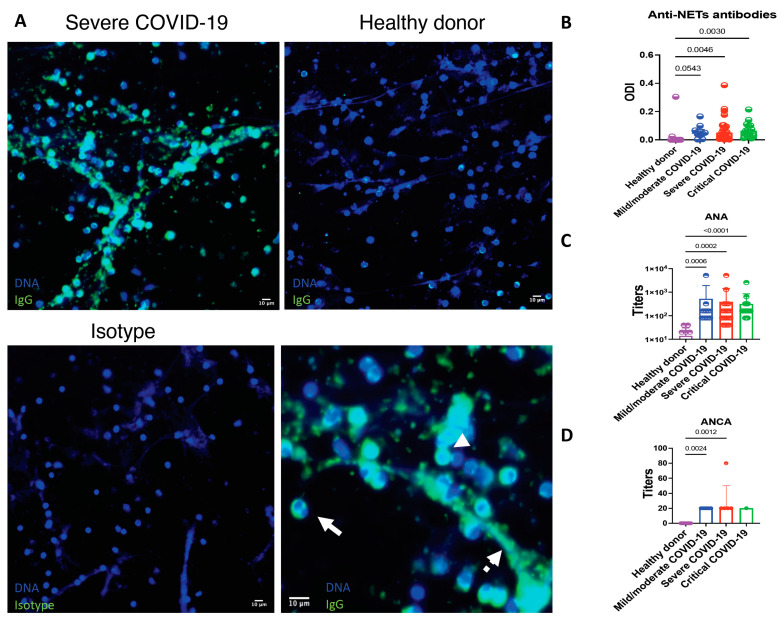
Patients with COVID-19 have antibodies that recognize neutrophils and NET’s antigens. (**A**) Healthy donor neutrophils were seeded on coverslips and stimulated with 2.5 mM PMA to induce the release of NETs. The samples were incubated with 10% sera from COVID-19 patients or healthy donors. Sera from COVID-19 patients show anti-neutrophil and anti-NET antibodies. The antigens recognized by anti-neutrophils and anti-NET antibodies include those located in the cytoplasm (continuous arrow), nucleus (arrowhead) and NETs (discontinuous arrow). The autoantibodies were not found in healthy donors. As the isotype control, the samples were incubated solely with the secondary antibody. (**B**) Relative amount of anti-NET antibodies expressed as the optic density index (ODI). (**C**) Titers of antinuclear antibodies expressed in a logarithmic scale. (**D**) Titers of anti-neutrophil cytoplasmic antibodies. Medians were compared using the Kruskal–Wallis and Dunn’s multiple comparison tests.

Enhanced formation of NETs has been related to the production of autoantibodies, therefore, we aimed to evaluate if patients with COVID-19 had anti-NET antibodies. In comparison to healthy donors, patients with COVID-19 had a higher prevalence of IgG anti-NET antibodies (30.00 vs. 68.50%), *p* = 0.019. There was not a difference in the prevalence or optic density index (ODI) of anti-NETs antibodies according to disease severity (Figure 5). As shown in Figure 5, the antibodies target antigens located in the nuclear and cytoplasmic compartments of neutrophils as well as in NETs. ANA and ANCA were evaluated in 66 and 20 patients, respectively. Patients with anti-NETs antibodies had a higher prevalence of positive ANAs (66.03% vs. 23.07%, OR 2.86, 95% CI 1.04–7.86, *p* = 0.006) and ANCAs (90.90% vs. 25.00%, OR 3.36, 95% CI 1.07–12.25, *p* = 0.006). The median (IQR) of ANA titers was 1:160 (1:80–1:320). The most frequent immunofluorescence ANA patterns were coarse speckled (33, 50.00%), cytoplasmic (25, 48.40%) and nucleolar (9, 13.63%). The median (IQR) of ANCA titers was 1:20 (1:20–1:20). The most common ANCA pattern was perinuclear (10, 50.00%). There was not a statistically significant difference in the titers of ANA and ANCA according to disease severity (Figure 5). ANA and ANCA above the positive cutoff were not detected in healthy donors.

LDG and NDG NETs from COVID-19 patients induce a proinflammatory response in monocyte-derived macrophages.

After we found an enhanced production and deficient degradation of NETs in patients with COVID-19, we aimed to assess if those NETs produce a differential cellular response in macrophages.

**Figure 6:**
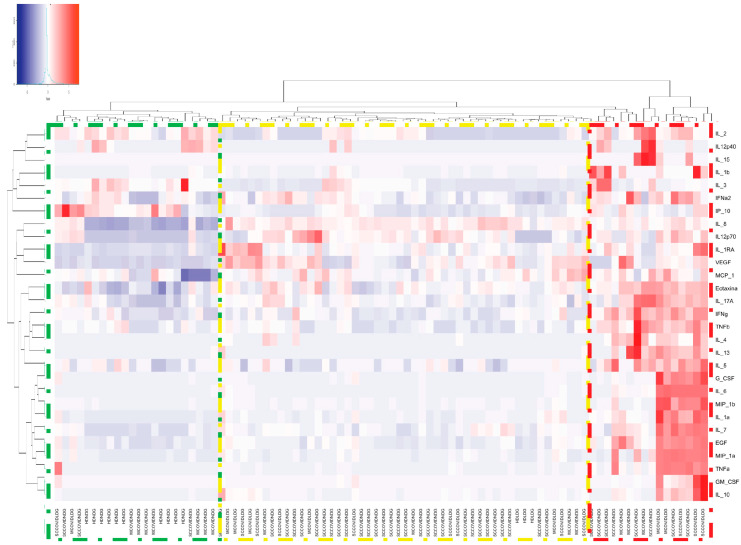
Heat map of the expression of cytokines and chemokines after macrophages were stimulated with COVID-19 and healthy donor NETs. The proinflammatory response is mainly elicited by COVID-19 LDG (red box) and NDG NETs (yellow box), whilst none of the healthy donor NETs promoted the secretion of these cytokines and chemokines (green box). Heatmap based on hierarchical clustering by Ward’s method was constructed.

In the heat map depicted in Figure 6, we detected three main clusters. In the first cluster (green box), NDG NETs from healthy donors were able to upregulate the expression of IL-2, IL-12p40 and IP-10. The second cluster (yellow box) is mainly composed of healthy donors LDG NETs and NDG NETs from severe/critical COVID-19 patients. These NETs were able to induce the expression of IL-8, IL-12p70, IL-1RA, VEGF and MCP-1. The third cluster (red box) included mostly LDG NETs from COVID-19 patients and is characterized by a robust upregulation of most of the cytokines and chemokines involved in COVID-19 pathogenesis (Table 3 and supplementary Figure S5), which suggest that LDG NETs from COVID-19 patients are highly proinflammatory.

## 5. Discussion

In this study, we found that patients with severe/critical COVID-19 have an enhanced production and deficient degradation of NETs, which carry a differential protein cargo and are mainly produced by NDG. NETs from COVID-19 LDG and NDG are able to induce a pro-inflammatory response in macrophages. Furthermore, patients with COVID-19 have anti-NET antibodies and this feature is related to the development of ANA and ANCA, highlighting their role in the inflammatory and autoimmune responses observed in COVID-19.

Previous studies have highlighted the importance of an enhanced granulocytic signature, including neutrophils similar to LDG [35] in peripheral blood of patients with severe COVID-19 [36]. Due to emergency myelopoiesis [36], neutrophils from these patients have a young immature activated phenotype demonstrated by their low expression of CD10 [37], CD16 and β-galactosidase as well as their enhanced degranulation [38]. Recent studies in patients with systemic lupus erythematosus (SLE) have shown that NDG and CD10^+^ LDG are more prone to NET formation in comparison with CD10^-^ LDG [39], which agrees with our results. The immature phenotype (CD10^-^) that we found in COVID-19 LDG, may explain their lower capacity to produce NETs. In this regard, immature neutrophils expressing PDL1 have been described in COVID-19 [36], which make COVID LDG more similar to myeloid derived suppressor cells.

Pro-inflammatory cytokines, immune complexes and microbial products are acknowledged enhancers of the production of NETs [10]. NDG from severe/critical COVID-19 patients may be primed *in vivo* to spontaneously produce NETs, since they are exposed to IL-6, IL-2, IL-7, TNF, CXCL10, MCP-1, MIP1a [40], GM-CSF and M-CSF during the cytokine storm [41]. Furthermore, bacterial products such as 16S rRNA and LPS have been detected in severe/critical COVID-19 patients even in the absence of bacterial infections [42] and it is known that patients with severe COVID-19 have decreased IgG fucosylation and increased levels of IgG3, IgM, and IgA [43], which promotes spontaneous release of NETs through the activation of the FcγR [10]. These *in vivo* stimuli may be responsible for the enhanced *ex vivo* production of NETs that we found.

In respiratory viral infections, NETs are both a defense mechanism and key mediators of lung damage. Previous studies have shown that SARS-CoV-2 triggers the secretion of NETs through the activation of the ACE2 receptor. Additionally, SARS-CoV-2 infects neutrophils, making them more prone to the release of NETs [29]. In this regard, NETs have been detected in many compartments of the lung, including the alveoli, interstitium, bronchi, and blood vessels [44], as well as in the liver and glomerulus of patients with COVID-19 [35].

NETs promote lung damage by inducing alveolar cell apoptosis [29] and increasing the density of mucus plugs [45]. In addition, there has been observed that ARDS patients with COVID-19 present necrotizing thrombotic lesions and pulmonary endotheliitis mediated by complement activation [46] and microvascular lesions induced by membrane attack complex (MAC) deposition [47]. Besides, NETs capture pro-thrombotic factors, including platelets, von Willebrand factor, tissue factor, histones, HMGB-1, neutrophil serine proteases, and fibrinogen whilst degrade anticoagulants such as thrombomodulin [48], favoring immunothrombosis.

A differential protein cargo of NETs is fundamental in the induction of tissue damage [10]. In this regard, NETs with tissue factor have been observed in patients with COVID-19 [48]. We found that COVID-19 NETs have a higher expression of HMGB1, LL-37 and ISG-15 in comparison with healthy donors. During viral infections, including COVID-19 [49], one of the most rapidly induced interferon related genes is ISG-15, which has been shown to inhibit viral replication, impede the externalization and latency of virions and, as an extracellular protein, functions as a chemotactic cytokine for neutrophils [50]. Accordingly, we found an increased expression of ISG-15 in NETs from pooled data of all included COVID-19 patients in comparison to healthy donors. Nonetheless, when we compared the amount of 1SG-15-DNA circulating complexes, we found lower levels of ISG-15-DNA complexes in severe COVID-19. These patients present an uncontrolled inflammatory response, similar to the type I IFN-mediated autoinflammation observed in humans with ISG-15 deficiency and to the cytokine storm observed in an ISG-15 deficient animal model of Chikungunya infection [50]. Patients with COVID-19 show a diminished type I Interferon signature in PBMCs, but neutrophils from these patients have an enhanced expression of interferon-related genes including *IFNα, IFIT1* and *ISG-15* [38] which agrees with our finding of a higher amount of ISG-15 expressed in NETs. Furthermore, it is known that the IFN response is time-dependent in COVID-19. A decrease in the type I IFN signature in patients with COVID-19 coincides with the progression to critical disease [51], which also supports our findings.

The antiviral effect of LL-37 has been demonstrated in influenza [52] and syncytial respiratory virus infections [53]. Nonetheless, the accumulation of LL-37 potentiates TLR3 signaling and the production of IL-6, IL-10 and MCP-1 in airway epithelial cells [54]. Therefore, the expression of LL-37 in NETs may contribute to the induction of the cytokine storm in COVID-19.

HMGB1 is externalized after the production of NETs and necrosis during hypoxia [55]. This alarmin acts as a pro-coagulant and promotes the secretion of TNF-α, IL-1β, IL-6 and IL-18 [55]. HMGB1 has been recently acknowledged as a critical regulator of the ACE2 expression and a modulator of SARS-CoV-2 entry into the cell [56] in agreement with our results.

We also found that sera from the critically ill and severe patients with COVID-19 have an impaired NET degradation capacity in serum, and such deficiency is reverted upon addition of recombinant human DNase. These data suggest an *in vivo* inhibition or a deficiency of endogenous DNase. In this regard, other viruses like type 5 Adenovirus have specific DNA-binding proteins that act as DNase inhibitors [57], and plasma levels of DNase-1 are markedly reduced in patients with SARS-CoV-2 sepsis [58]. DNase-1 coated long-acting nanoparticles have been suggested as a therapeutic intervention to efficiently reduce the accumulation of NETs and the NF-κB-induced cytokine production in a mouse model of sepsis by improving the stability of the enzyme [58]. Another beneficial effect of DNase is its potential role in the viscoelasticity of respiratory tract secretions, decreasing the severity of COVID-19 and improving lung function [59]. Besides, the administration of dornase alfa in COVID-19 patients has shown to improve *in vitro* NETs and viral clearance, even 72 h after the appearance of the first symptom [59]. Further studies are necessary to unveil if SARS-CoV-2 proteins inhibit DNase activity.

Along with neutrophils, macrophages are the main inflammatory infiltrate in viral lung infections [60]. In bronchoalveolar lavage fluid (BALF) and peripheral blood of patients with COVID-19 there is an enrichment of genes related to monocytes, macrophages and neutrophils [49], particularly in severe patients [61]. Likewise, *IL1A, IL1B, IL1R2, IL1RN, IL18, IL6, TNF, IL10,* and *TGFB1* are highly expressed by monocyte-macrophages from BALF [49]. Our study highlights that internalization of NETs by macrophages results in the production of pro-inflammatory cytokines. Therefore, an enhanced production, deficient degradation and a differential protein cargo in COVID-19 NETs may be key drivers of the cytokine storm induced by macrophages.

In COVID-19, some studies have suggested the role of molecular mimicry, bystander activation, epitope spreading and cryptic antigen presentation in the expansion of autoimmune damage [62]. The constant activation of monocytes, macrophages, and neutrophils involving an enhanced release of NETs during viral infections such as COVID-19, induces chromatin reorganization through histone acetylation or methylation, which has been shown to impair the normal host tolerance to microbes and self-antigens [63].

The breach in peripheral tolerance characterized by the production of diverse autoantibodies was recently described as a hallmark of critical COVID-19 [24]. Accordingly, we found an increased frequency of positive anti-NET antibodies in COVID-19 patients compared to healthy controls. Other reports have described that ANA induction is associated with a somber prognosis in COVID-19, including complications during hospitalization and death [64]. According to our data, the increased charge of chromatin, DNA and histones that are externalized during the production of NETs is known to promote the production of anti-NET antibodies, which are related to ANA positivity [65]. Autoantibodies against extracellular and secreted proteins in COVID-19 patients are highly prevalent and are associated with disease severity [65]. In accordance with our results, the presence of ANA, anti-Ro, anti-La, anti-U1-RNP, anti-centromere and anti-Scl-70 antibodies have been observed in patients with COVID-19, regardless of disease severity [66]. Furthermore, previous studies have reported positive ANCA in patients with COVID-19 [67], although their clinical significance is unknown. This is the first study to associate the presence of ANA and ANCA with anti-NET antibodies. There is an enhancement of extrafollicular B cell responses in COVID-19 patients and in autoimmune settings like SLE. This response is driven by double negative B cells and expanded by TLR7 ligands, including SARS-CoV-2, explaining, at least partly, the autoreactive antibodies against the NETs components [21].

Our study has several limitations, including the biased analyses of the protein cargo of the NETs. Since we were not able to perform MS analyses, we cannot rule out the role of other proteins in the cellular responses to NETs in SARS-CoV-2 infection. Although the detection of plasmatic protein–DNA complexes is an acknowledged marker of circulating NETs, these complexes may also be released as part of other types of cell death like necrosis [68]. Nonetheless, we were able to corroborate that LL-37, HMGB1 and ISG-15 are expressed in NETs from COVID-19 patients by confocal microscopy. Another limitation is that we only assessed ANA and ANCA in a limited number of patients and we do not know the pre-COVID19 serological status of those subjects. Finally, since previous studies have already described the diminished DNase-1 levels in patients with COVID-19, we only aimed to assess the functional defect of this trait in the degradation capacity of NETs. We acknowledge that one of the main limitations of our study is that we did not measure the serum levels of DNase-1 nor its activity. Nonetheless, our study unveils a mechanism by which the previously described low levels of DNase-1 may be harmful in patients with COVID-19 and supports its use as a therapeutic agent for this disease.

Graphic abstract. Patients with severe and critical COVID-19 have a higher amount of immature LDG, which correlates with markers of hypoxia, tissue damage, immunothrombosis, serum cytokines and circulating NETs. Both NDG and LDG from patients with COVID-19 produce spontaneous NETs with a pro-inflammatory protein cargo that promotes the secretion of pro-inflammatory cytokines in macrophages. Sera from severe and critical COVID-19 patients have a deficient NET degradation capacity, which is restored after addition of hrDNase. The enhanced production of NETs in COVID-19 patients is a driver of hyperinflammation and autoimmunity, since anti-NET antibodies are related to ANA and ANCA positivity.

## Figures and Tables

**Table 1:** Proportion of LDG subsets and plasmatic NET complexes in healthy donors and COVID-19 patients according to disease severity.

Variable	Healthy Donors Median (IQR) *n* = 10	Mild/Moderate Median (IQR) *n* = 27	Severe Median (IQR) *n* = 27	Critical Median (IQR) *n* = 28	*p*-Value
Low Density Granulocytes (LDG)					
Total LDG (%)	0.46 (0.31–0.68)	0.59 (0.34–1.23)	2.35 (0.97–4.43)	12.75 (3.19–18.43)	<0.001
Total LDG (cells/mm^3^)	23 (15.75–34.13)	42.33 (19.1–99.79)	181.00 (55.29–307.20)	1109 (332.10–4080.00)	<0.001
CD10 + LDG (%)	0.13 (0.05–0.22)	0.05 (0.01–0.10)	0.20 (0.07–0.21)	0.156 (0.30–12.63)	<0.001
CD10 + LDG (cells/mm^3^)	0.02 (0.01–0.06)	0.02 (0.00–0.03)	0.39 (0.05–5.06)	18.98 (1.18–323.50)	<0.001
CD10 − LDG (%)	0.31 (0.17–0.46)	0.52 (0.31–1.00)	1.65 (0.61–3.32)	5.98 (2.22–11.53)	<0.001
CD10 − LDG (cells/mm^3^)	0.07 (0.02–0.15)	0.22 (0.05–0.84)	2.54 (0.45–6.64)	106.5 (7.2–225.7)	<0.001
Circulating Plasmatic NETs					
HMGB1-DNA complexes (ODI)	0.5 (0.35–0.55)	0.45 (0.32–0.62)	0.59 (0.44–0.87)	1.57 (0.98–2.49)	<0.001
ISG-15-DNA complexes (ODI)	0.50 (0.40–0.53)	0.69 (0.58–1.07)	0.61 (0.47–0.76)	0.81 (0.68–0.97)	<0.001
LL-37-DNA complexes (ODI)	0.33 (0.30–0.85)	1.03 (0.52–1.30)	1.12 (0.75–2.49)	0.93 (0.69–1.05)	0.05
NE-DNA (NETs) complexes (ODI)	0.58 (0.38–1.72)	0.83 (0.49–1.23)	1.39 (0.86–1.73)	1.11 (0.85–1.68)	0.001

ODI: optic density index.

**Table 2:** Amount of NETs and their protein cargo measured by confocal microscopy.

Variable	Healthy Donor LDG (*n* = 3)	Healthy Donor NDG (*n* = 10)	COVID-19 LDG (*n* = 20)	COVID 19 NDG (*n* = 41)	*p*
NETs (%)	0.60 (0.50- 1.00)	0.78 (0.46–1.13)	3.44 (1.75–5.83)	2.04 (1.22–3.20)	<0.001
NETs length (µm)	1.00 (0.45–2.00)	2.48 (0.44–3.38)	8.22 (2.65–15.78)	27.15 (14.80–41.80)	<0.001
MFI HMGB1	7.4 (0.49–11.2)	6.39 (3.00–12.85)	35.21 (27.94–39.95)	30.58 (24.75–43.16)	<0.001
MFI LL-37	7.46 (1.60–21.30)	8.48 (0.00–18.51)	36.58 (28.16–46.80)	52.78 (37.05–58.74)	<0.001
MF1 ISG-15	11.00 (0.00–22.00)	8.48 (0.00–18.51)	54.51 (30.23–85.34)	85.02 (50.59 113.10)	<0.001

MFI: mean fluorescent intensity.

**Table 3:** Differential expression of cytokines and chemokines in supernatant after macrophage stimulation with NETs.

Variable	HD NDGs *n* = 10	HD LDGs *n* = 3	Mild/Moderate COVID-19 NGDs *n* = 14	Mild/Moderate COVID-19 LDGs *n* = 8	Severe/Critical COVID-19 NDGs *n* = 41	Severe/Critical COVID-19 LDGs *n* = 12	*p*
EGF (pg/mL)	6.41 (2.85–8.23)	3.50 (1.46–6.98)	6.12 (1.46–19.94)	18.11 (14.05–53.53)	10.27 (6.7–16.48)	12.73 (7.98–60.14)	0.003
Eotaxin (pg/mL)	11.70 (9.18–13.94)	16.26 (15.00–16.26)	16.90 (14.19–20.80)	18.43 (15.94–22.29)	17.13 (14.91–19.31)	17.17 (14.23–20.06)	0.01
G-CSF (pg/mL)	2.25 (2.25–2.97)	1 (0.68–2.25)	24.27 (2.25–69.95)	89.39 (24.95–3923.00)	25.43 (8.97–52.13)	79.21 (25.99–6976.00)	<0.001
IFN-α (pg/mL)	15.1 (11.27–17.27)	5.00 (4.28–6.00)	10.88 (2.19–15.65)	10.95 (8.96–13.97)	15.10 (8.27–17.28)	12.49 (7.35–19.36)	0.076
IFN-γ (pg/mL)	1.56 (0.66–2.49)	2.67 (2.00–3.01)	2.28 (1.33–3.98)	2.66 (2.13–5.21)	2.32 (1.56–4.38)	2.28 (1.56–5.80)	0.35
IL-10 (pg/mL)	1.95 (0.55–3.93)	9.00 (8.32–10.46)	13.98 (3.97–36.09)	92.85 (21.69–180.5)	23.83 (7.39–41.98)	54.21 (22.01–284.50)	<0.001
IL12 p40 (pg/mL)	0.40 (0.40–9.35)	0.40 (0.40–0.40)	0.40 (0.40–5.05)	0.40 (0.40–0.40)	0.40 (0.40–0.40)	0.40 (0.40–0.40)	0.19
IL-12 p70 (pg/mL)	1.02 (1.02–1.02)	1.02 (1.02–1.02)	3.16 (1.02–4.56)	5.65 (2.72–10.93)	6.76 (4.06–9.71)	7.78 (4.28–8.95)	<0.001
IL-13 (pg/mL)	0.32 (0.32–0.32)	0.32 (0.32–0.32)	0.32 (0.32–0.49)	0.32 (0.32–2.04)	0.32 (0.32–0.32)	0.32 (0.32–1.85)	0.37
IL-15 (pg/mL)	0.25 (0.25–0-25)	0.25 (0.25–0-25)	0.25 (0.25–0-25)	0.25 (0.25–0-25)	0.25 (0.25–0-25)	0.25 (0.25–0-25)	0.79
IL-17A (pg/mL)	0.76 (0.36–1.37)	1.04 (1.00–1.31)	1.57 (0.09–2.38)	1.45 (1.31–3.63)	1.31 (0.76–1.95)	2.44 (1.36–2.90)	0.05
IL-1RA (pg/mL)	30.99 (22.17–44.24)	76.97 (50.00–100.80	96.80 (30.41–220.60)	187.80 (86.50–393.30)	142.00 (96.65–208.20)	184.00 (104.50–591.10)	<0.001
IL-1A (pg/mL)	0.60 (0.60–1.95)	8.00 (7.52–10-62)	10.36 (1.70–20.89)	24.13 (16.04–185.10)	17.93 (11.69–35.22)	29.52 (13.16–206-90)	<0.001
IL-1b (pg/mL)	6.42 (4.80–9.67)	17.00 (16.65–18.90)	19.23 (17.57–54.05)	35.83 (17.33–84.03)	25.16 (17.90–81.10)	20.43 (13.76–175.20)	<0.001
1L-2 (pg/mL)	2.75 (2.50–0.91)	0.06 (0.06–0.06)	0.55 (0.47–0.83)	0.12 (0.06–0.53)	0.48 (0.06–0.72)	0.17 (0.06–0.50)	0.006
IL-3 (pg/mL)	0.95 (0.64–1.40)	0.24 (0.24–0.26)	0.45 (0.28–0.49)	0.26 (0.21–0.37)	0.48 (0.20–0.61)	0.23 (0.18–0.52)	0.001
IL-4 (pg/mL)	1.12 (1.12–1.12)	1.12 (1.12–1.12)	1.12 (1.12–8.94)	1.12 (1.12–7.65)	1.12 (1.12–1.12)	1.12 (1.12–3.78)	0.304
IL-5 (pg/mL)	0.59 (0.51–0.68)	0.59 (0.59–0.59)	0.59 (0.59–0.59)	0.63 (0.53–0.74)	0.59 (0.51–0.68)	0.59 (0.53–0.85)	0.973
IL-6 (pg/mL)	12.66 (6.05–20.03)	9.33 (9.00–10.30)	23.20 (14.20–104.60)	86.09 (29.33–1675.00)	27.97 (19.36–56.99)	49.31 (23.91–1673.00)	<0.001
IL-7 (pg/mL)	1.00 (1.00–1.00)	3.30 (3.00–4.88)	6.34 (2.86–9.52)	7.87 (5.05–26.34)	6.66 (4.43–11.81)	6.19 (4.72–28.92)	<0.001
IL-8 (pg/mL)	658.20 (434.70–1998-00)	9183.00 (6000.00–11775.00)	10,002.00 (1146.00–11110.00)	10,361.00 (9594.00–11901.00)	10,561.00 (9134.00–12112.00)	10,619.00 (9433.00–11711.00)	<0.001
IP-10 (pg/mL)	139.70 (103.00–178.60)	45.00 (41.47–52.03)	70.88 (55.77–134.20)	69.84 (49.78–82.74)	59.39 (46.70–96.74)	75.52 (51.32–171.00)	0.0291
MCP-1 (pg/mL)	4549.00 (4121.00–4989.00)	5000.00 (4662.00–6092.00)	5070.00 (2748.00–6557.00)	4970.00 (4256.00–6078.00)	4942.00 (4263.00–5525.00)	4561.00 (4287.00–5983.00)	0.654
MIP-1α (pg/mL)	15.02 (5.82–68.16)	60.00 (54.71–73.83)	68.29 (27.73–82.28)	279.00 (165.70–2117.00)	146.90 (123.00–528.10)	280.70 (120.30–2157.00)	<0.001
MIP-1β (pg/mL)	0.12 (0.12–159.50)	56.96 (50.00–66.36)	58.56 (15.05–81.45)	454.20 (304.10–6673.00)	213.80 (133.00–533.10)	281.90 (169.70–4616.00)	<0.001
TNF-α (pg/mL)	17.33 (9.21–58.63)	40.00 (39.60–42.15)	53.02 (16.11–386.70)	499.20 (127.30–7598.00)	217.60 (62.71–741.00)	417.30 (156.30–10032)	<0.001
TNF-β (pg/mL)	0.65 (0.39–0.94)	0.50 (0.39–0.65)	0.32 (0.11–0.71)	0.65 (0.42–1.10)	0.39 (0.11–0.65)	0.53 (0.39–1.26)	0.399
VEGF (pg/mL)	22.19 (14.37–29.62)	50.00 (49.54–68.10)	148.70 (15.54–213.70)	193.40 (139.10–236.00)	88.00 (28.48–140.90)	178.10 (135.60–232.20)	<0.001
GM-CSF (pg/mL)	1.63 (0.72–4.88)	0.60 (0.13–0.73)	1.90 (0.85–2.66)	7.18 (1.62–42.50)	3.38 (1.63–18.06)	5.17 (2.15–343.70)	0.006

## Data Availability

All data generated or analyzed during this study is included in this published article and its Supplementary Material.

## Supplementary Materials

The following are available online at https://www.mdpi.com/article/10.3390/cells10102545/s1, Figure S1: Proportion of the subsets of low-density granulocytes and amount of circulating NETs according to COVID-19 severity, Figure S2: Correlations between the LDG subsets and the inflammatory and severity markers of COVID-19, Figure S3: Clinical features of patients with SARS-CoV-2 infection according to COVID-9 severity, Figure S4: Serum levels of cytokines and chemokines according to the COVID-19 severity, Figure S5: Cytokines and chemokines secreted by macrophages from healthy donors after stimulation with NETs, Table S1: Clinical features of healthy donors and patients with COVID-19 according to disease severity, Table S2: Serum cytokine and chemokine levels of patients with COVID-19 according to disease severity.
