# Supplementary material for: Neutrophil Extracellular Traps Contribute to COVID-19 Hyperinflammation and Humoral Autoimmunity
Source: Cells. 2021 Sep 26;10(10):2545. doi: 10.3390/cells10102545 (PMC8533917; doi:10.3390/cells10102545)
Supplement: Supplementary file 1 [file cells-10-02545-s001.zip › cells-1356384-supplementary.pdf]

Supplementary material

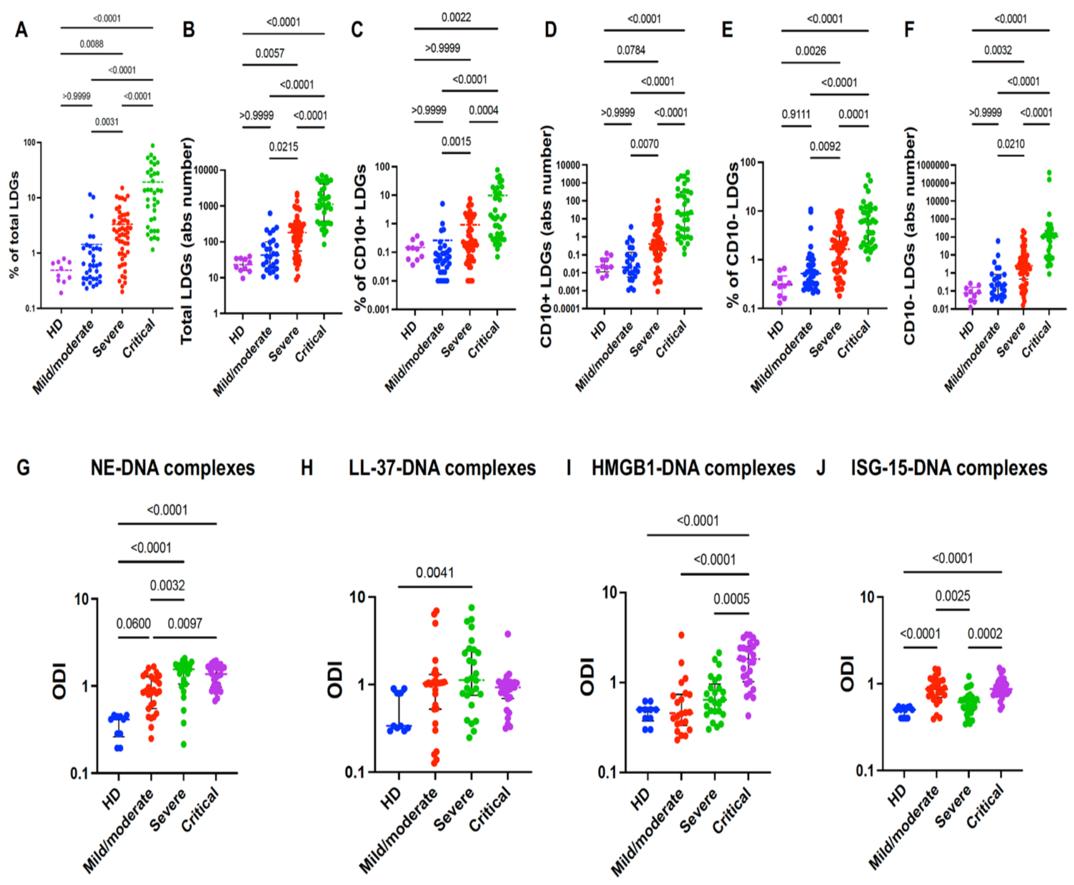

Figure S1. Proportion of the subsets of low-density granulocytes and amount of circulating NETs according to COVID-19 severity. A-F. The proportion and absolute number of low-density granulocytes augment according to disease severity. Immature CD10^{-} LDG predominate in patients with COVID-19. G-J. Patients with critical COVID-19 have a higher amount of plasmatic neutrophil elastase (NE)-DNA, HMGB1-DNA and ISG-15-DNA complexes. Subjects with severe COVID-19 show an increased plasmatic concentration of LL-37-DNA complexes and a lower amount of ISG-15-DNA complexes. All the dot plots are shown in a logarithmic scale. The amount of circulating plasmatic NETs is expressed as the optic density index (ODI). Medians were compared with the Kruskal-Wallis and the Dunn's tests for multiple comparisons.

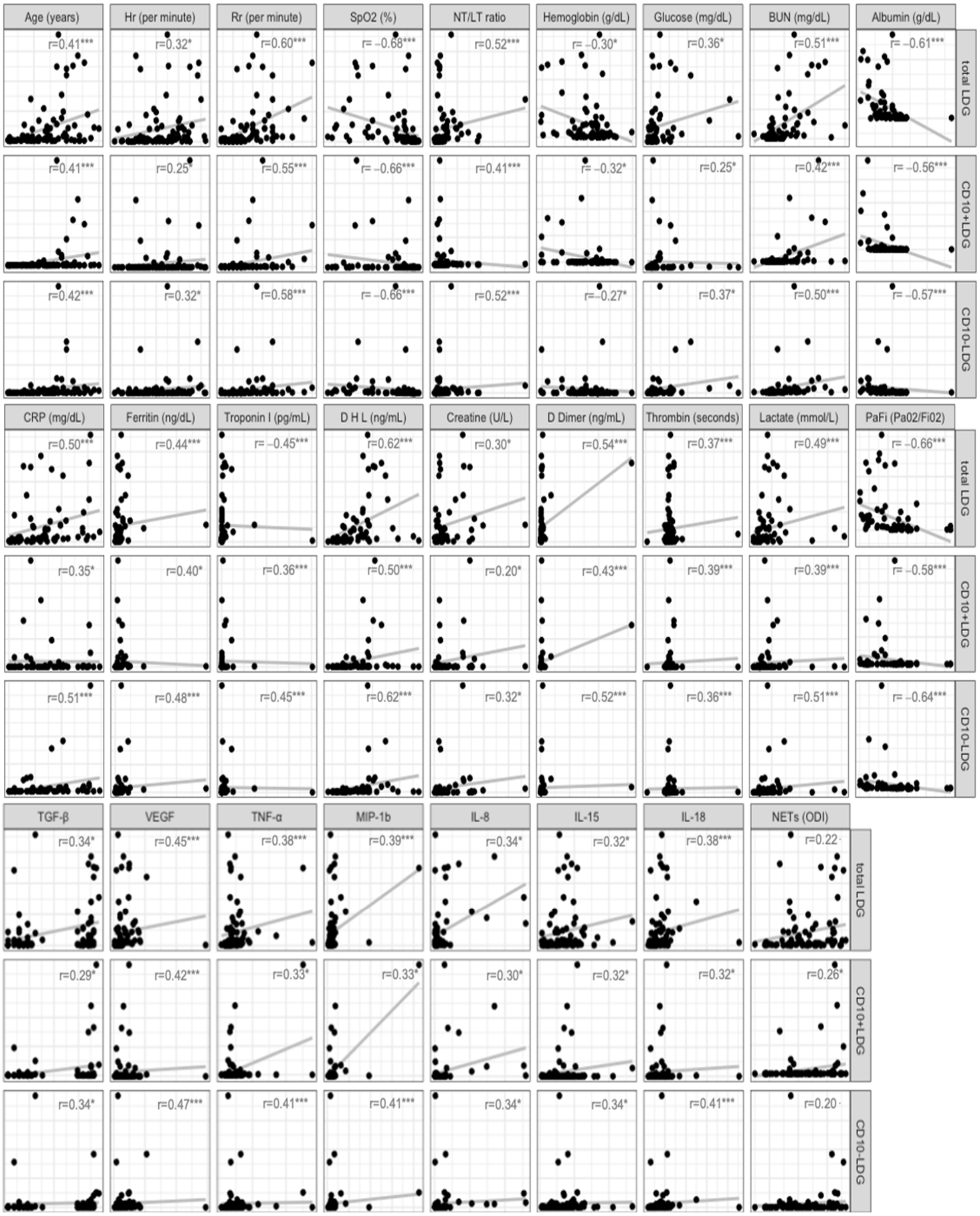

Figure S2. Correlations between the LDG subsets and the inflammatory and severity markers of COVID-19.

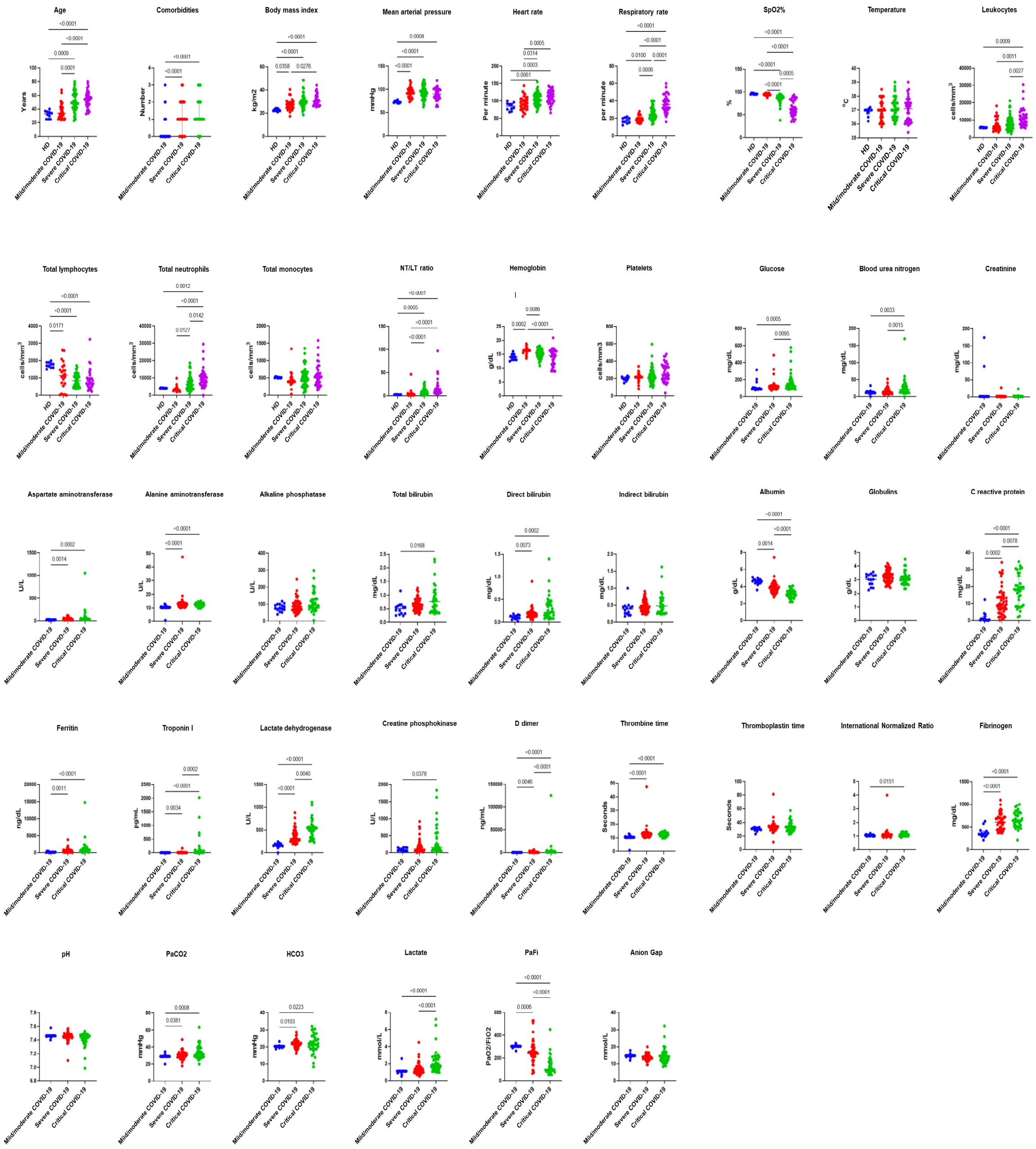

Figure S3. Clinical features of patients with SARS-CoV-2 infection according to COVID-9 severity. Differences between medians were addressed with the Kruskal-Wallis and the Dunn's tests for multiple comparisons.

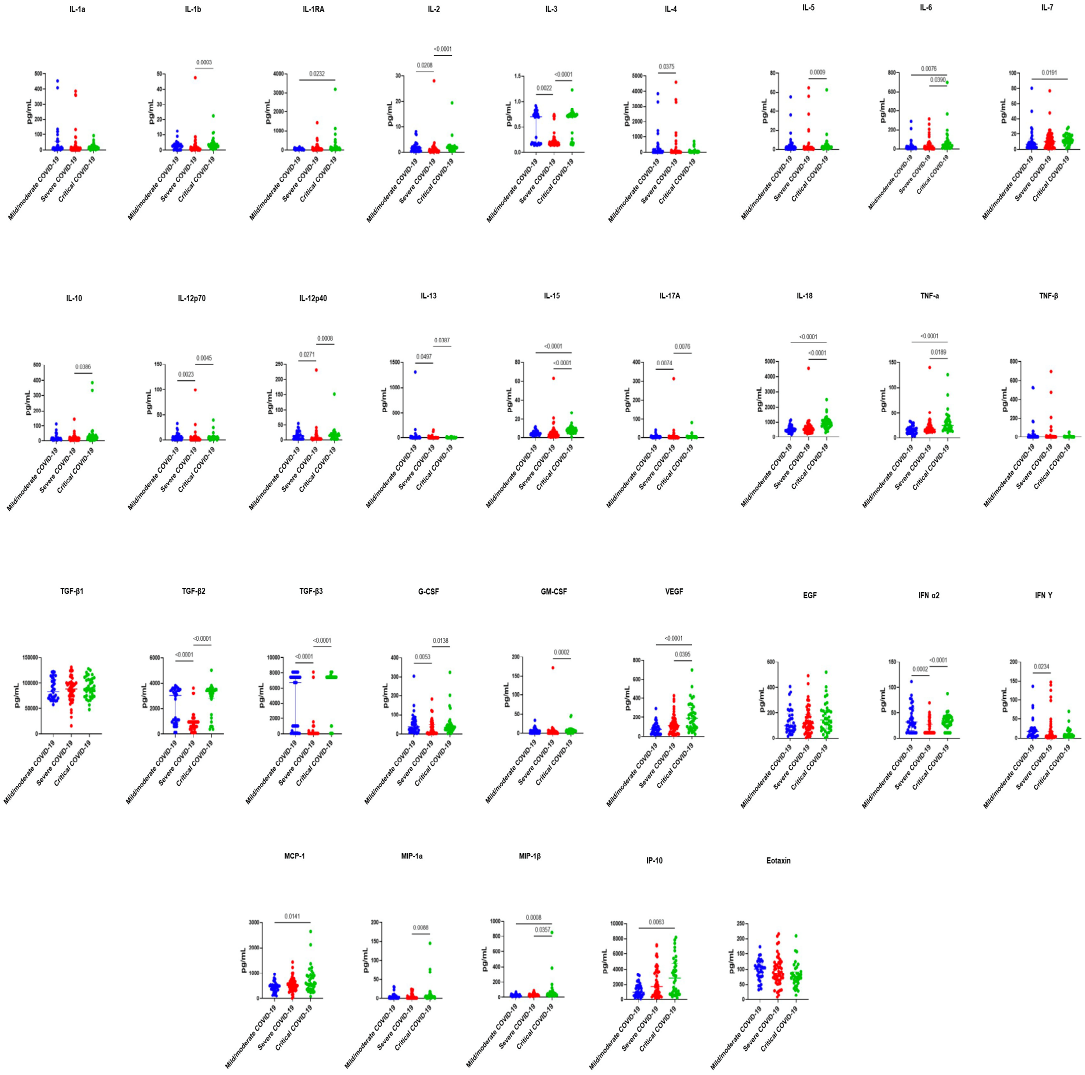

Figure S4. Serum levels of cytokines and chemokines according to the COVID-19 severity. Medians were compared with the Kruskal-Wallis and Dunn's tests for multiple comparisons.

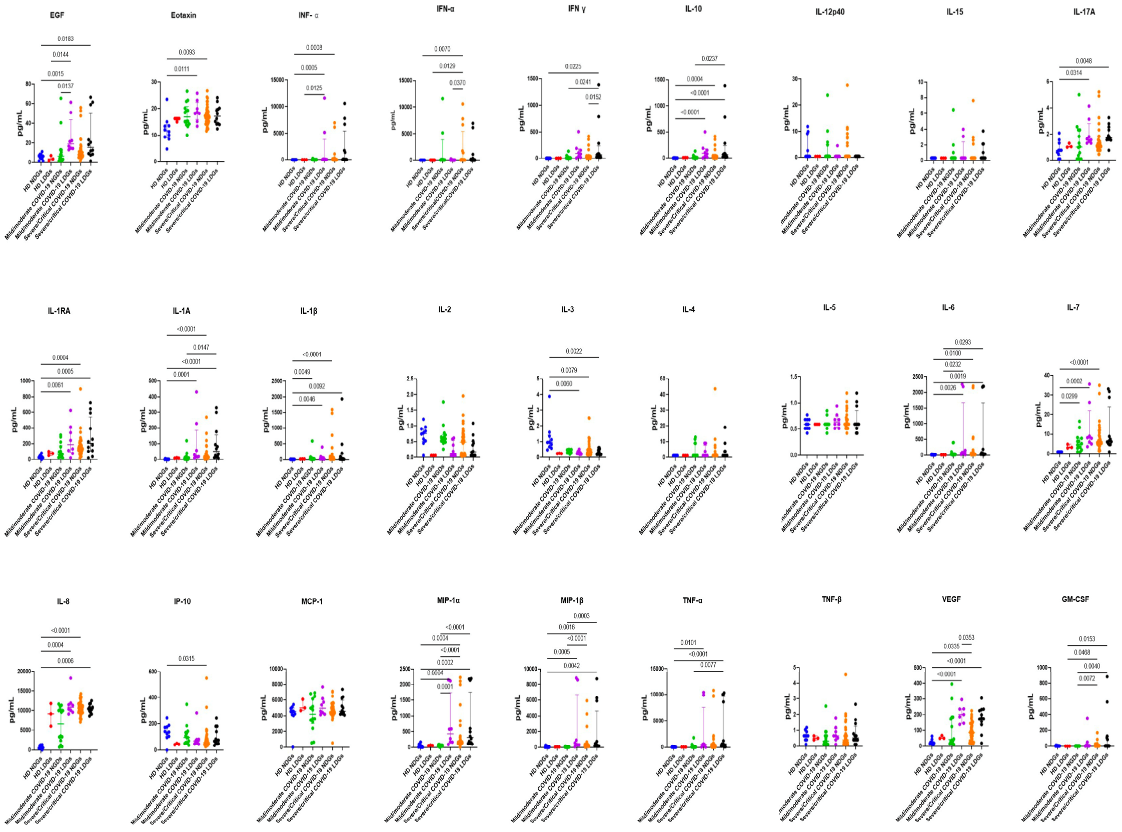

Figure S5. Cytokines and chemokines secreted by macrophages from healthy donors after stimulation with NETs. Two million macrophages were stimulated with 50 mcg of NETs for 6 hours. The levels of cytokines and chemokines were addressed in the supernatants. Differences between medians were assessed with the Kriskal-Wallis and Dunn’s tests for multiple comparisons.

Table S1. Clinical features of healthy donors and patients with COVID-19 according to disease severity.

Variable	Healthy donors Median (IQR) N=10	Mild/moderate Median (IQR) N=27	Severe Median (IQR) N=27	Critical Median (IQR) N=28	*P*-value
**Demographic features**					
Male N (%)	6 (60.00%)	16 (59.25%)	18 (66.66%)	24 (85.71%)	-
Age (years)	33.00 (25.00-36.25)	35.00 (27.50-42.50)	48.00 (40.50-60.50)	56.00 (47.75-63.25)	<0.001
Comorbidities (Number)	0 (0-0)	0.00 (0.00-0.00)	1.00 (0.00-2.00)	1.00 (1.00-2.00)	<0.001
**Clinical features**					
Body mass index (kg/m^2)	23.00 (22.00-24.00)	26.80 (24.25-29.20)	29.50 (27.25-31.21)	30.10 (25.65-34.38)	0.011
Mean arterial pressure (mmHg)	73.00 (70.75-78.00)	93.00 (86.00-100.00)	93.00 (78.30-104.50)	86.00 (47.75-65.50)	0.636
Heart rate (beats per minute)	85.00 (74.50-93.00)	94.00 (81.50-104.50)	105.00 (92.50-118.50)	111.50 (96.75-128.25)	0.008
Respiratory rate (per minute)	17.00 (14.75-20.25)	18.00 (17.00-22.00)	24.00 (22.00-29.50)	33.50 (30.00-40.00)	<0.001
(SpO2, %)	96.00 (95.00-97.25)	95.00 (94.00-95.00)	88.00 (85.00-91.00)	66.00 (51.50-83.25)	<0.001
Temperature (^{o}C)	37.00 (36.73-37.03)	37.00 (36.50-37.55)	37.30 (37.00-37.50)	36.80 (36.17-37.50)	0.111
**Laboratory parameters**					
Leukocytes (cells/mm^3)	5700 (5575-5825)	7650.00 (5200.0-16275.00)	7700.00 (5300.00-10450.00)	10700.00 (8075.00-13075.00)	0.058
Total lymphocytes (cells/mm^3)	1750 (1600-1900)	1436.00 (1059.00-1487.77)	676.00 (505.00-1052.00)	809.50 (583.00-1192.80)	<0.001
Total neutrophils (cells/mm^3)	4161 (4070-4252)	3715.00 (3501.00-3715.00)	6100.00 (4338.00-8568.00)	9158.00 (6895.00-11934.00)	<0.001
Total monocytes (cells/mm^3)	513 (501.80-524.30)	395.00 (395.00-401.80)	462.00 (311.00-583.50)	532.50 (387.00-663.5)	0.035
NT/LT ratio	2.40 (2.24-2.57)	2.50 (2.17-3.12)	9.35 (5.95-13.04)	10.51 (6.35-15.81)	<0.001
Hemoglobin (g/dL)	14.00 (13.00-15.00)	16.35 (16.35-16.35)	15.60 (14.20-16.80)	14.75 (11.60-16.12)	<0.001
Platelets (cells/mm^3)	200 (185.00-220.00)	217.80 (217.80-217.80)	215.00 (189.50-270.00)	238.00 (194.80-333.00)	0.162
Glucose (mg/dL)		101.00 (96.00-107.00)	107.00 (102.00-125.50)	146.5 (111.00-208.50)	<0.001
Blood Urea Nitrogen (mg/dL)		13.80 (12.50-33.00)	14.00 (10.80-19.65)	23.35 (14.97-35.52)	0.005

Creatinine (mg/dL)		0.97 (0.79-1.07)	0.95 (0.77-1.15)	0.99 (0.78-1.23)	0.693
Aspartate aminotransferase (U/L)		23.10 (20.30-32.00)	37.00 (29.00-62.00)	47.90 (32.92-89.33)	<0.001
Alanine aminotransferase (U/L)		3.00 (1.00-6.00)	40.00 (22.85-52.30)	39.45 (31.45-63.58)	0.166
Alkaline phosphatase (U/L)		78.00 (69.00-93.00)	99.00 (67.00-116.50)	97.50 (80.75-130.00)	0.264
Total bilirubin (mg/dL)		0.56 (0.45-0.60)	0.65 (0.50-0.75)	0.77 (0.43-1.04)	0.093
Direct bilirubin (mg/dL)		0.10 (0.09-0.14)	0.19 (0.15-0.23)	0.23 (0.15-0.43)	<0.001
Indirect bilirubin (mg/dL)		0.42 (0.36-0.46)	0.46 (0.34-0.51)	0.48 (0.27-0.72)	0.418
Albumin (g/dL)		4.63 (4.40-4.78)	3.63 (3.39-4.19)	3.24 (2.81-3.55)	<0.001
Globulins (g/dL)		3.02 (2.70-3.35)	3.22 (2.92-3.40)	3.08 (2.79-3.27)	0.364
C reactive protein (mg/dL)		1.16 (0.45-2.40)	10.94 (6.91-13.81)	19.29 (13.62-28.02)	<0.001
Ferritin (ng/dL)		7.00 (6.00-25.00)	449.00 (253.50-661.50)	845.00 (458.00-1326.20)	<0.001
Troponin I (pg/mL)		1.60 (1.2-1.8)	5.5 (3.5-6.5)	14.80 (6.35-89.00)	<0.001
Lactate dehydrogenase (U/L)		179.00 (156.00-200.00)	312.00 (280.00-422.00)	542.50 (460.20-612.80)	<0.001
Creatine phosphokinase (U/L)		115.00 (65.00-154.00)	141.00 (50.50-230.00)	152.00 (97.25-492.25)	0.216
D Dimer (ng/mL)		259.00 (195.00-351.00)	496.00 (417.00-789.50)	1324.00 (1054.00-2400.00)	<0.001
Thrombin time (Seconds)		10.40 (10.40-10.45)	12.90 (12.05-13.60)	11.95 (11.50-13.30)	<0.001
Thromboplastin time (Seconds)		29.90 (29.90-30.05)	32.70 (30.40-34.50)	32.75 (28.70-39.00)	*<0.001*
International Normalized Ratio		1.06 (1.06-1.06)	1.10 (1.00-1.20)	1.10 (1.00-1.20)	0.043
Fibrinogen (mg/dL)		364.50 (332.80-413.00)	603.00 (450.00-714.00)	685.00 (522.00-816.20)	<0.001
**Arterial blood gases**					
pH		7.46 (7.46-7.46)	7.47 (7.44-7.48)	7.43 (7.380-7.46)	0.003
PaCO2 (mmHg)		28.92 (28.92-28.92)	30.30 (28.35-31.90)	31.30 (28.93-39.12)	0.001
HCO3 (mmHg)		20.34 (20.34-20.34)	21.80 (20.30-22.75)	21.40 (18.95-24.27)	0.011
Lactate (mmol/L)		1.14 (1.14-1.14)	1.20 (1.00-1.54)	1.80 (1.40-2.52)	<0.001
PaFi		304.80 (304.80)	255.00 (219.50-306.50)	109.50 (86.50-166.80)	<0.001
Anion Gap (mmol/L)		14.70 (14.70-14.70)	13.70 (12.85-15.15)	14.20 (11.93-16.75)	*0.194*
Days since symptoms started		3.00 (2.00-5.75)	9.00 (7.00-14.00)	7.00 (5.00-11.00)	<0.001

Table S2. Serum cytokine and chemokine levels of patients with COVID-19 according to disease severity.

Variable	Mild/moderate Median (IQR) N=27	Severe Median (IQR) N=27	Critical Median (IQR) N=28	*P*-value
IL-1a (pg/mL)	13.94(7.21-57.00)	9.44(1.93-24.00)	18.38(11.22-25.42)	0.174
IL-1b (pg/mL)	2.54(0.38-3.80)	0.62(0.36-1.81)	3.29(2-27-4.66)	<0.001
IL-1RA (pg/mL)	51.51(34.92-79.75)	48.32(21.79-63.24)	98.67(41.87-168.86)	0.025
IL-2 (pg/mL)	1.38(0.40-2.05)	0.64(0.36-1.32)	1.86(1.54-2.07)	<0.001
IL-3 (pg/mL)	0.29(0.16-0.75)	0.18(0.16-0.21)	0.72(0.70-0.74)	<0.001
IL-4 (pg/mL)	62.58(11.90-245.31)	11.90(11.90-76.02)	50.96(40.26-67.91)	0.065
IL-5 (pg/mL)	2.5(0.45-4.68)	0.85(0.60-2.12)	2.51(1.94-4.30)	0.033
IL-6 (pg/mL)	14.57(9.30-26.64)	23.29(7.42-54.68)	41.07(18.53-7.34)	0.027
IL-7 (pg/mL)	6.85(2.50-11.61)	10.11(6.94-16.88)	12.11(9.24-17.48)	0.035
IL-8 (pg/mL)	14.06(8.89-22.26)	19.11(9.13-41.72)	51.26(31.60-174.19)	<0.001
IL-10 (pg/mL)	15.00(10.14-24.61)	15.30(8.37-20.61)	26.38(13.46-36.65)	0.088
IL-12p70 (pg/mL)	4.49(0.59-7.90)	0.59(0.59-2.52)	4.70(3.58-5.59)	<0.001
IL-12p40 (pg/mL)	10.66(2.95-23.27)	2.95(2.95-4.12)	14.49(9.87-17.62)	0.004
IL-13 (pg/mL)	6.21(0.56-15.56)	0.56(0.56-3.55)	3.42(2.89-4.45)	0.026
IL-15 (pg/mL)	4.47(3.28-6.27)	3.46(1.28-7.19)	8.35(6.68-10.33)	<0.001
IL-17A (pg/mL)	5.04(2.09-6.81)	1.18(0.60-3.16)	4.86(3.30-7.06)	0.003
IL-18 (pg/mL)	534.50(444.20-677.20)	553.6(360.90-734.30)	968.5(794.3-1015.40)	<0.001
TNF-α (pg/mL)	15.79(10.70-20.51)	19.00(15.43-26.07)	24.88(17.70-32.90)	0.001
TNF-β (pg/mL)	7.41(0.70-24.63)	0.70(0.70-5.8)	3.44(267.00-4.04)	0.072
TGF- β1 (pg/mL)	79954.00(67056.00-97678.00)	87206(74317-99132)	85937.00(73119.00-105938.00)	0.436
TGF-β2 (pg/mL)	1092.50(764.10-3407.70)	764.1(481.2-932.9)	3407.70(2963.90-3515.80)	<0.001
TGF-β3 (pg/mL)	1023.00(238.61-744.03)	68.24(68.24-68.24)	7444.03(744.03-744.03)	<0.001
G-CSF (pg/mL)	36.18(18.89-75.31)	3.87(3.87-34.33)	37.10(25.69-49.88)	<0.001
GM-CSF (pg/mL)	5.31(0.50-8.86)	0.50(0.50-3.30)	6.70(5.44-9.19)	<0.001
VEGF (pg/mL)	60.26(30.70-93.17)	127.99(93.54-186.98)	176.76(98.75-244.81)	<0.001
EGF (pg/mL)	93.65(57.88-152.24)	121.65(93.58-165.17)	129.89(76.38-216.85)	0.171
IFN-α2 (pg/mL)	29.11(10.90-58.66)	10.90(10.90-14.40)	33.41(23.82-42.93)	<0.001
IFN-γ (pg/mL)	17.82(7.18-25.91)	6.24(30.5-17.34)	8.31(5.65-20.89)	0.122
MCP-1/CCL2 (pg/mL)	501.80(353.40-537.40)	506.39(422.92-723.10)	616.34(381.68-909.06)	0.057
MIP-1α/CCL4 (pg/mL)	3.29(0.70-7.54)	0.78(0.70-5.28)	5.01(3.49-8.08)	0.015

MIP-1β/CCL3 (pg/mL)	28.30(16.95-37.88)	30.82(22.61-37.70)	48.30(29.19-69.10)	0.003
IP-10/CXCL10 (pg/mL)	1414.80(644.90-1849.10)	1766.40(923.90-3610.90)	3193.80(900.80-4934.90)	0.017
Eotaxin/CCL11 (pg/mL)	97.20(74.94-118.93)	80.11(60.34-113.75)	74.28(59.56-95.33)	0.213
